# Effect of γ-aminobutyric acid producing bacteria on *in vitro* rumen fermentation, growth performance, and meat quality of Hanwoo steers

**DOI:** 10.5713/ajas.19.0785

**Published:** 2020-01-13

**Authors:** Lovelia L. Mamuad, Seon Ho Kim, Min Jung Ku, Sang Suk Lee

**Affiliations:** 1 Ruminant Nutrition and Anaerobe Laboratory, College of Bio-industry Science, Sunchon National University, Suncheon 57922, Korea; 2 Livestock Research Institute, Jeonnam Agricultural Research and Extension Services, Gangjin 59213, Korea

**Keywords:** Antioxidant, Biogenic Amines, γ-Aminobutyric Acid, Hanwoo Steers, *In vitro* Rumen Fermentation

## Abstract

**Objective:**

The present study aimed to evaluate the effects of γ-aminobutyric acid (GABA)-producing bacteria (GPB) on *in vitro* rumen fermentation and on the growth performance and meat quality of Hanwoo steers.

**Methods:**

The effects of GPB (*Lactobacillus brevis* YM 3-30)-produced and commercially available GABA were investigated using *in vitro* rumen fermentation. Using soybean meal as a substrate, either GPB-produced or commercially available GABA were added to the *in vitro* rumen fermentation bottles, as follows: control, no additive; T1, 2 g/L GPB; T2, 5 g/L GPB; T3, 2 g/L autoclaved GPB; T4, 5 g/L autoclaved GPB; T5, 2 g/L GABA; and T6, 5 g/L GABA. In addition, 27 Hanwoo steers (602.06±10.13 kg) were subjected to a 129-day feeding trial, during which they were fed daily with a commercially available total mixed ration that was supplemented with different amounts of GPB-produced GABA (control, no additive; T1, 2 g/L GPB; T2, 5 g/L GPB). The degree of marbling was assessed using the nine-point beef marbling standard while endotoxin was analyzed using a Chromo-*Limulus* amebocyte lysate test.

**Results:**

In regard to *in vitro* rumen fermentation, the addition of GPB-produced GABA failed to significantly affect pH or total gas production but did increase the ammonia nitrogen (NH_3_-N) concentration (p<0.05) and reduce total biogenic amines (p<0.05). Animals fed the GPB-produced GABA diet exhibited significantly lower levels of blood endotoxins than control animals and yielded comparable average daily gain, feed conversion ratio, and beef marbling scores.

**Conclusion:**

The addition of GPB improved *in vitro* fermentation by reducing biogenic amine production and by increasing both antioxidant activity and NH_3_-N production. Moreover, it also reduced the blood endotoxin levels of Hanwoo steers.

## INTRODUCTION

γ-Aminobutyric acid (GABA) is a major inhibitory neurotransmitter in the mammalian central nervous system that has other known physiological functions, including etiotropic effects on the health status and growth rates of calves [1] and protective effects against neurotoxicant-induced cell death [2]. The GABA is considered as a potent bioactive compound [3] that is synthesized through the irreversible α-decarboxylation of L-glutamic acid, which is catalyzed by glutamic acid decarboxylase [2]. In recent years, researchers have primarily focused on the production of GABA by lactic acid bacteria (LAB) because it possess special physiological activities that could also be used as functional starters [4]. In addition, the production of GABA by LAB is considered safe and eco-friendly [5]. Some LAB species can produce high concentrations of GABA, and the most commonly used bacteria in GABA production are *Lactococcus lactis*, *Lactobacillus brevis*, *Lb. paracassei*, and *Lb. delbrüeckii* subsp. *bulgaricus* [6]. One of these GABA-producing bacteria (GPB), namely *Lb. brevis*, was isolated from kimchi and produces GABA that may inhibit or regulate certain biogenic amine (BA) compounds, such as histamine and tyramine [7].

Owing to the potential of GPB to be used in animal feeding, the present study was conducted to evaluate the ability of the GPB *Lb. brevis* YM 3-30 to reduce BAs and increase antioxidative substances, such as superoxide dismutase (SOD) and glutathione peroxidase (GSH-Px), using an *in vitro* fermentation technique. *In vitro* gas production has been used as a valuable tool for evaluating the nutritional value of feedstuffs [8] and techniques for mitigating methane production among ruminants [9]. In the present study, soybean meal, which is a by-product of soybean oil extraction and the most important source of high-quality vegetable protein in animal feed, was used as a fermentation substrate. Furthermore, an *in vivo* feeding trial was conducted to determine the blood endotoxin level and performance of Hanwoo steer provided with GPB-produced GABA-supplemented feed, in terms of average daily gain (ADG), feed conversion ratio (FCR), and marbling score.

## MATERIALS AND METHODS

### GPB preparation and *in vitro* experimental design

The feeding trial on animal growth performance was conducted at the Sunchon National University (SCNU) animal farm while the laboratory experiments was conducted at the Ruminant Nutrition and Anaerobe Laboratory, Department of Animal Science and Technology, SCNU, Jeonnam, South Korea that were reviewed and approved by the Sunchon National University Animal Research Ethics Committee (SCNU IACUC, approval number: SCNU IACUC-2012-04).

The fermentation processes that occur inside the rumen were simulated using an *in vitro* ruminal fermentation technique. The GPB *Lb. brevis* YM 3-30 was isolated from kimchi and anaerobically cultivated in deMan, Rogosa, and Sharpe (MRS) broth (Becton-Dickinson and Company, Difco, Sparks, MD, USA; pH 5.0) that was supplemented with 5% monosodium glutamate (MSG) at 32°C for 48 h. The GPB had an optical density of 1.8 at 600 nm, which corresponded to 10^7^ colony-forming unit (CFU)/mL and a GABA concentration of 45 mg/mL. Using soybean meal as a substrate, either GPB-produced or commercially available GABA were added to the *in vitro* rumen fermentation bottles, as follows: control, no additive; treatment 1 (T1), 2 g/L GPB; treatment 2 (T2), 5 g/L GPB; treatment 3 (T3), 2 g/L autoclaved GPB; treatment 4 (T4), 5 g/L autoclaved GPB; treatment 5 (T5), 2 g/L GABA; treatment 6 (T6), and 5 g/L GABA. Fresh GPB culture was added to treatments 1 and 2, whereas autoclaved (121°C for 15 min) GPB was added to treatments 3 and 4, and commercially available GABA (99.9%; Sigma-Aldrich, Ltd., St. Louis, Mo, USA) was added to treatments 5 and 6.

### *In vitro* rumen fermentation

Ruminal contents were collected from three 48-month-old ruminally cannulated Hanwoo steers with body weights of 600±47 kg. The contents were squeezed, and the extracted fluids were strained through cheesecloth that had been folded four times. The extracted and strained fluids were then transferred to a glass bottle and maintained at 39°C in a water bath. The upper residue of the rumen fluid was removed using a vacuum pump, whereas the middle portion was collected and used in the experiment. The pooled, particle-free rumen fluid was transferred to a buffer medium [10] (pH 6.7) that was prepared according to the method described by Russell and Van Soest [10].

Next, the buffered rumen fluid (50 mL) was anaerobically transferred under a constant flow of CO_2_ gas to 160-mL serum bottles that contained soybean meal (2% dry matter [DM] basis; particle size: 2 mm). The bottles that contained the mixed substrate and buffered rumen fluid were sealed using rubber stoppers and aluminum caps and were incubated at 39°C for 12, 24, or 48 h in a shaking incubator (100 rpm). Three replicates were performed for each treatment and incubation period, and the total gas (TG), pH, ammonia nitrogen (NH_3_-N), BA, SOD, and GSH-Px were analyzed after each incubation period. Duplicates of one ml sample were also collected from each of the serum bottles and kept at −50°C until analyses for volatile fatty acids (VFAs) and NH_3_-N.

The TG production was measured in each of the serum bottles using a press and sensor machine (Laurel Electronics, Inc., Costa Mesa, CA, USA). After TG measurement, the bottles were uncapped, and the pH of each bottle was measured using a Pinnacle series M530p meter (Schott Instruments, Mainz, Germany). Rumen fluid was collected, transferred to Eppendorf tubes, centrifuged, and the NH_3_-N concentration of the resulting supernatant was measured using the methods developed by Chaney and Marbach [11]. Meanwhile, the VFAs and other metabolites were measured using high-performance liquid chromatography (HPLC; Agilent Technologies 1200 series, Waldbronn, Baden-Wuttemberg, Germany), according to the methods described by Han et al [12] and Tabaru et al [13], and BA concentrations were measured using Waters Liquid Chromatography (Waters Ltd., Milford, MA, USA) with a Varian column (Pursuit × Rs 5u C-18 250 × 4.6 mm; Varian, Inc., Palo Alto, CA, USA) [7]. Finally, total SOD activity was determined by measuring the inhibition of pyrogallol autoxidation (with and without sample) while the GSH-Px was determined by the presence of reduced glutathione and hydrogen peroxide as described in our previous study [7].

### Animals and experimental design

Twenty-seven 24- to 25-month-old Hanwoo steers, with an average initial weight of 602.06±10.13 kg, were subjected to a 129-day feeding trial. The animals were randomly selected and equally distributed among the following three groups: control, T1, and T2. During the experiment, the animals were housed in separate pens. The initial weights were measured upon commencement of the feeding trial (day 1), whereas final weight measurement and blood collection for endotoxin analysis were conducted at the termination of the feeding trial (day 129).

### Diet and feeding

Each steer was fed daily with 13 kg commercially available total mixed ration. The feed was divided into morning and afternoon feeding. The feed and chemical composition was shown in Table 1. The GPB *Lb. brevis* YM 3-30 was cultivated using MRS broth (Becton, Dickinson and Company, Sparks, MD, USA; pH 5.0) that was supplemented with 5% MSG at 32°C or 48 h, and fresh GPB broth cultures with similar bacterial densities and GABA concentrations were added to the animals’ feed. The GABA produced by GPB were evaluated and these were: control, T1, T2. To evenly distribute the GPB within the feed, the amount of GPB to be fed to each animal was mixed into 200 g of either soybean meal (first 60 d) or wheat bran (last 69 d) prior to feeding, and the GPB were added to the feed by sprinkling the inoculated mixtures onto the TMR offered during the morning.

### Growth performance and meat quality

The weight gain of the experimental animals was calculated as the difference between initial weight, which was measured on day 1, and the final weight, which was measured on day 129, whereas ADG was calculated by dividing the weight gain by 129, and FCR was calculated as the ratio of DM intake (DMI) to ADG. Experimental animals were slaughtered after completion of the feeding trial, at which time the degree of marbling was assessed, using the nine-point beef marbling standard, which is regularly used to estimate the quality of Korean beef carcasses. In this grading system, which was established by the Korea Institute of Animal Products Quality Evaluation, a score of 9 indicates very abundant marbling, whereas a score of 1 indicates little or no marbling [14].

### Analysis of *in vivo* plasma endotoxins

For the determination of the plasma endotoxin levels, 3 mL blood was extracted from each animal, by tail venipuncture, on the last day of the feeding trial (day 129) and then centrifuged. The resulting plasma (1.5 mL) was transferred to a microtube and sent to Woojung Bio Co., Ltd. Inc. (Suwon, Korea) for endotoxin analysis, using a Chromo-LAL (*Limulus* amebocyte lysate) test. Briefly, co-lyophilized LAL and substrate reagent were mixed with a test sample in a microplate and incubated in a reader at 37°C±1°C. Absorbance measurements were then collected at various times following the addition of Chromo-LAL and analyzed. The time (onset time) required for a sample to reach a specified absorbance (onset optical density) was calculated, after which a standard curve that represented the linear correlation between log onset time and log concentration of standard endotoxin was generated. The maximum range of endotoxin concentrations for the standard curve was 0.005 to 50 EU/mL, and the maximum sensitivity (λ) of the assay was defined as the lowest concentration used in the standard curve (0.005 EU/mL).

### Statistical analysis

Data were analyzed by analysis of variance using the general linear model for randomized complete block design. All treatments in *in vitro* and *in vivo* studies were conducted in three and nine replications, respectively, and Duncan’s multiple range test was used to identify differences between specific treatments. A significance value of p<0.05 was considered to indicate statistical significance. All analyses were carried out using Statistical Analysis Systems (SAS) version 9.4 [15].

## RESULTS

### *In vitro* rumen fermentation of GABA producing bacteria

As shown in Table 2, pH decreased significantly as incubation time increased, with the lowest pH being obtained at 24 and 48 h. Meanwhile, TG production and NH_3_-N concentration exhibited increasing trends from 12 to 48 h of incubation (p<0.05). The TG produced after 48 h of incubation by the GABA in T6 tended to be higher than other treatments, however, did not differ significantly from that of the control, whereas NH_3_-N concentration was lowest in the control (p<0.05) and greatest in the T6 treatment (p<0.05), at levels of 107.80 and 158.20 mg/dL, respectively. However, the treatments failed to significantly affect the concentrations of acetate, propionate, or butyrate or the acetate:propionate ratio (Table 3). Notably, higher total VFA production at 48 h in non-autoclaved GPB-produced GABA than autoclaved GPB-produced GABA. Furthermore, the greatest total VFA production at 48 h (108.31 mM) was observed in the T6 treatment and was 11.63 mM greater than that of the control (p<0.05).

**Figure 1:**
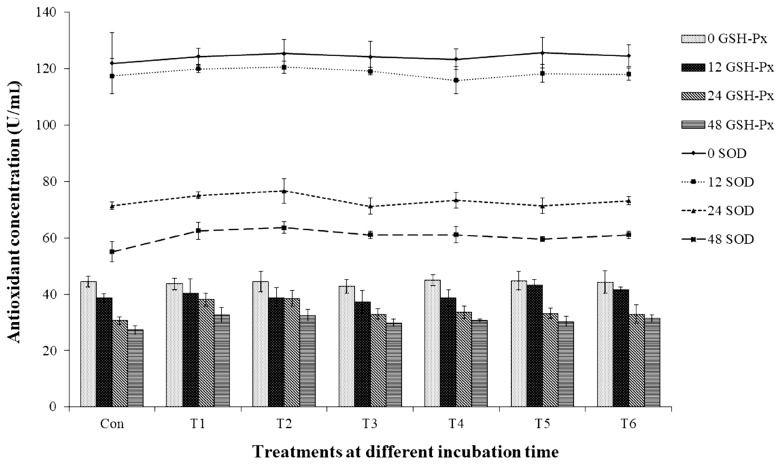
Effect γ-aminobutyric acid on the antioxidant concentrations of *in vitro* fermentation. Con, 0 g/L GPB; T1, 2 g/L GPB; T2, 5 g/L GPB; T3, 2 g/L autoclaved GPB; T4, 5 g/L autoclaved GPB; T5, 2 g/L GABA; and T6, 5 g/L GABA. GABA, γ-aminobutyric acid; GPB, GABA-producing bacteria; GSH-Px, glutathione peroxidase; SOD, superoxide dismutase. Values and error bars represent mean±standard deviation values (n = 3).

The total BA (Table 4) was highest after 48 h of incubation, and the control group yielded the highest concentration (17.22 mM), whereas the treatment groups that contained non-autoclaved GPB-produced GABA and GABA (T1, T2, T5, and T6) yielded the lowest concentrations (10.62 to 11.21 mM; p<0.05). In addition, the control group produced the most histamine (15.99 mM). In contrast, treatment failed to significantly affect the production of SOD or GSH-Px, even though the groups without GPB-produced GABA or GABA exhibited the lowest SOD and GSH-Px levels (55.06 and 27.32 U/mL, respectively). As shown in Figure 1, antioxidant enzyme levels decreased over time, with the highest levels of both SOD and GSH-Px being observed after 48 h of incubation (p<0.05).

### Effect of GABA producing bacteria on Hanwoo steers

As shown in Table 5, the Hanwoo steers fed with GPB-produced GABA exhibited superior weight performance than the control animals. The mean weight gains of the T1 and T2 groups were 6.50 and 18.34 kg greater than those of the control group, respectively, which resulted in higher, but not significantly so, ADG values in the T1 (0.76 kg) and T2 (0.85 kg) groups than in the control (0.71 kg). Treatment also failed to affect the marbling score of the meat. However, as shown in Table 5, the T1 and T2 groups exhibited lower blood endotoxin levels (17.23 and 16.42 EU/mL, respectively) than the control group (29.23 EU/mL; p<0.05).

## DISCUSSION

### *In vitro* rumen fermentation

The significant effects of GABA treatment on pH were only observed during the first 12 h of incubation, and then pH remained relatively stable for the next 36 h, as previously reported by Lounglawan and Suksombat [16], who found that the ruminal pH of dairy cows was not affected by either *Lb. plantarum* or *Lb. acidophilus* supplementation at 1×10^9^ CFU/cow/d, when administered with 200 g/d soybean oil. The similar pH values of the control and GABA-treated groups in the present study suggest that the addition of GPB or GABA did not make the *in vitro* fermentation more acidic and, therefore, that the treatment would not reduce either fiber digestion or the number of fiber-degrading bacteria, both of which are negatively affected by low pH. In addition, the GPB used in the present study was a LAB that could elicit its mode of action as a direct-fed microbe through stabilization of ruminal pH [17] and, therefore, lessen the occurrence of ruminal acidosis.

The concentration of NH_3_-N also increased with incubation duration, which indicated N became available for microbial utilization and protein synthesis as the incubation period progressed. The NH_3_-N concentrations of the experimental rumen fluid were considered sufficient for the maximum growth of rumen microbes, whereas a minimum of ~80 mg N/L was required to achieve maximum carbohydrate degradation [18]. The addition of GPB failed to significantly affect the production of individual VFAs, which confirms the findings of Raeth-Knight et al [19], who reported that supplementing the diet of Holstein dairy cows with *Lb. acidophilus* and *Propionibacteria freudenreichii*, did not affect ruminal fluid characteristics, at least in terms of individual and total VFA production. However, it was observed in this study that the non-autoclaved GPB-produced GABA had higher efficiency than autoclaved GPB-produced GABA by having higher total VFA production.

The apparently lower concentration of histamine and total BA in the GABA-treated groups than in the control indicates the positive effect of including GPB. This finding was unexpected since the addition of GPB-produced GABA tends to produce more GABA because LAB, such as *Lb. brevis*, decarboxylate amino acids from substrates to form BAs. Moreover, lower individual and total BA concentrations in the non-autoclaved GPB-produced GABA indicate higher efficiency than autoclaved GPB-produced GABA. *In vivo* studies conducted by Buchanan-Smith [20] revealed that the addition of BAs and GABA to a silage basal diet can reduce intake owing to the possible role of nitrogenous constituents. However, Dawson and Mayne [21] reported that amines, GABA, or silage juice, at concentrations of ≤2 g/kg, either added directly to the diet or administered *via* intraruminal infusion had no significant effect on the voluntary food intake of steer. A relationship between histamine concentration and hyperacidity during metabolic acidosis has also been reported by a variety of functional studies of non-ruminant gastrointestinal cuticles [22]. Moreover, Aschenbach and Gäbel [23] concluded that promotion of systemic acidosis by histamine absorption is due to luminal acidity-induced ruminal epithelial damage and not to histamine.

The SOD and GSH-Px are the primary oxygen free radical-eliminators that decrease during stressful environmental conditions. LeBlanc et al [24], who evaluated the anti-inflammatory effects of catalase (CAT)- or SOD-producing LAB on mice using a trinitrobenzenesulfonic acid-induced Crohn’s disease murine model, reported that mice fed CAT- or SOD-producing LAB exhibited more rapid recoveries from initial weight loss, increased enzymatic activities in the gut, and less intestinal inflammation. Zhang et al [25] also reported that dietary GABA can improve antioxidation among heat-stressed laying hens, as indicated by a significant increase in SOD and GSH-Px activity.

### Effect of GABA producing bacteria on Hanwoo steers

Hanwoo steers fed GPB-produced GABA exhibited superior weight performance than the control animals, even though the difference was not statistically significant. Similar findings were reported by Cruywagen et al [26], who reported that supplementing a milk substitute with *Lb. acidophilus* did not significantly affect feed efficiency among pre-weaned calves that were older than 2 weeks of age. These findings suggest that animals will benefit most from GPB-produced GABA when supplementation is conducted during the early growth stages. Moreover, Ando et al [27] reported that the addition of LAB (*Lb. plantarum* and *Lb. rhamnosus* NGRI 0110) improved silage quality and increased both digestibility and voluntary intake. In addition, treatment did not affect the deposition of intramuscular fat, as indicated by the similar marbling scores of treated and control animals in this study.

It is unlikely that the lower plasma endotoxin levels of the steers fed GPB-produced GABA can be attributed to the reductions in rumen lipopolysaccharide (LPS) and histamine levels caused by GPB-produced GABA supplementation since plasma concentrations of LPS and histamine should not be significantly affected by rumen contents [28]. However, studies in rats suggest that the attenuation of bacteremia and endotoxemia by *Lactobacilli* administration could be partly attributed to enhanced intestinal motility [29] and protection of the liver against LPS-induced injury, both of which are thought to contribute to the systemic clearance and detoxification of LPS, *via* anti-oxidative and anti-inflammatory effects [30].

## CONCLUSION

The present study demonstrates the potential utility of GPB in the development of feed additives. In the *in vitro* fermentation experiments, the inclusion of GPB-produced GABA (2 g/L and 5 g/L) yielded the greatest reduction in BAs and greatest increase in NH_3_-N production and antioxidant activity. Moreover, the supplementation of cattle feed with GPB reduced plasma endotoxin levels. However, the possible role of the GPB in increasing NH_3_-N concentration and reducing BA production *in vitro* and reducing plasma endotoxin level *in vivo* has yet to be established, and the effects of GABA treatment on rumen microorganisms should be studied further to elucidate the mechanisms underlying the effects of GPB probiotics in cattle.

## Figures and Tables

**Table 1:** Total mixed ration feed and chemical composition

Items	As basis %
Ingredients	
Basal feed	39.10
Brewer’s grain	14.00
Soy sauce cake	18.50
Rice straw	3.10
Italian ryegrass	20.00
Alfalfa pellet	5.00
Yeast	0.30
Basal feed	
Corn	49.50
Molasses	3.00
Tapioca	2.50
Wheat bran	9.00
Corn gluten feed	9.00
Soybean meal	6.00
Rapeseed oil meal	6.00
Coconut meal	6.00
Palm kernel cake	6.00
Salt and vitamin premix1)	3.00
Chemical composition (% dry matter)	
Dry matter	71.90
Crude protein	11.12
Crude fat	2.60
Crude fiber	9.44
Ash	8.63
Calcium	1.76
Phosphorus	0.39

1) Vitamin premix contained the following amount which was diluted in cellulose (g/kg premix): L-ascorbic acid, 121.2; DL-atocopherol acetate, 18.8; thiamin hydrochloride, 2.7; riboflavin, 9.1; pyridoxine hydrochloride, 1.8; niacin, 36.4; Ca-D-pantothenate, 12.7; myo-inositol, 181.8; D-biotin, 0.27; folic acid, 0.68; p-aminobenzoic acid, 18.2; menadione, 1.8; retinal acetate, 0.73; cholecalciferol, 0.003; cyanocobalamin, 0.003.

**Table 2:** Effect of γ-aminobutyric acid on the pH, gas production, and ammonia concentration of *in vitro* fermentation

Time (h)	Treatment1)							Mean	p-value

	Con	T1	T2	T3	T4	T5	T6		
pH									
12	5.61^c^	5.64^abc^	5.64^abc^	5.65^ab^	5.68^a^	5.66^ab^	5.63^bc^	5.64^w^	<0.001
24	5.49	5.45	5.46	5.48	5.50	5.45	5.45	5.46^x^	
48	5.42	5.41	5.43	5.41	5.42	5.44	5.42	5.48^x^	
Total gas production (mL)									
12	74.00^b^	75.00^b^	80.33^a^	82.67^a^	83.00^a^	71.00^b^	71.67^b^	76.62^x^	<0.001
24	77.00	81.00	80.33	78.33	79.67	77.00	79.00	79.10^x^	
v48	96.50^ab^	91.00^b^	99.67^ab^	99.67^b^	102.33^ab^	98.33^ab^	108.00^a^	99.43^w^	
NH_3_-N concentration (mg/dL)									
12	75.55^c^	79.00^bc^	5.00^abc^	90.73^ab^	96.47^a^	74.93^c^	79.90^bc^	83.06^y^	<0.001
24	96.10^d^	104.10^c^	104.07^c^	104.33^c^	103.57^c^	105.43^b^	110.13^a^	103.93^x^	
48	107.75^c^	127.67^b^	130.67^b^	128.6.33^b^	117.07^bc^	118.70^bc^	158.20^a^	127.11^w^	

GABA, γ-aminobutyric acid.

1) Con, 0 g/L GPB; T1, 2 g/L GPB; T2, 5 g/L GPB; T3, 2 g/L autoclaved GPB; T4, 5 g/L autoclaved GPB; T5, 2 g/L GABA; and T6, 5 g/L GABA.

Different superscript letters within rows (^a–d^) and columns (^w–z^) indicate significant differences at the 5% level, as indicated by Duncan’s multiple range test.

**Table 3:** Effect γ-aminobutyric acid on volatile fatty acid production during *in vitro* fermentation

Time (h)	Treatment1)							Mean	p-value

	Con	T1	T2	T3	T4	T5	T6		
Acetate (mM)									
12	44.92	41.43	44.03	42.11	44.23	43.02	42.70	43.21	0.412
24	41.98	45.86	43.56	43.55	44.01	45.75	44.13	44.12	
48	49.30^ab^	49.15^ab^	50.67^ab^	48.61^ab^	46.66^b^	51.90^a^	52.47^a^	49.82	
Propionate (mM)									
12	16.43^a^	14.77^b^	15.35^ab^	15.19^ab^	15.98^ab^	15.47^ab^	15.78^ab^	15.57	0.024
24	16.81	17.43	17.45	18.96	18.95	17.72	18.28	17.94	
48	22.20	23.98	23.07	26.61	23.85	23.75	24.96	24.06	
Butyrate (mM)									
12	8.59^d^	9.67^abcd^	10.83^a^	10.36^ab^	10.10^abc^	9.46^bcd^	8.91^cd^	9.70	0.056
24	13.48	12.92	15.41	14.18	16.44	15.08	13.26	14.39	
48	25.19^ab^	29.62^a^	27.37^ab^	21.61^b^	21.14^b^	21.40^b^	30.88^a^	25.31	
Acetate:propionate ratio (mM)									
12	2.73	2.80	2.87	2.77	2.77	2.78	2.71	2.78	<0.001
24	2.50^ab^	2.63^a^	2.50^ab^	2.30^b^	2.32^b^	2.58^a^	2.41^ab^	2.46	
48	2.22	2.05	2.20	1.83	1.96	2.19	2.10	2.08	
Total volatile fatty acids (mM)									
12	69.94^cd^	65.87^d^	70.21^a^	67.65^cd^	70.31^ab^	67.95^cd^	67.39^bc^	68.47	0.473
24	72.27^d^	76.21^cd^	76.42^abcd^	76.68^abc^	79.40^a^	78.55^ab^	75.67^bcd^	76.46	
48	96.68^c^	102.75^b^	101.11^b^	96.84^c^	91.65^d^	97.04^c^	108.31^a^	99.20	

GABA, γ-aminobutyric acid; GPB, GABA-producing bacteria.

1) Con, 0 g/L GPB; T1, 2 g/L GPB; T2, 5 g/L GPB; T3, 2 g/L autoclaved GPB; T4, 5 g/L autoclaved GPB; T5, 2 g/L GABA; and T6, 5 g/L GABA.

Different superscript letters within rows (^a–d^) indicate significant differences at the 5% level, as indicated by Duncan’s multiple range test.

**Table 4:** Effect γ-aminobutyric acid on the concentration of biogenic amines during *in vitro* fermentation

Time (h)	Treatment1)							Mean	p-value

	Con	T1	T2	T3	T4	T5	T6		
Histamine (mM)									
12	14.16^a^	12.13^ab^	11.01^bc^	11.43^bc^	10.55^bc^	10.08^bc^	9.66^c^	11.29	<0.001
24	14.72^a^	9.41^b^	10.42^b^	11.19^b^	10.95^b^	9.98^b^	9.36^b^	10.86	
48	15.99^a^	9.34^c^	9.96^c^	11.51^b^	13.23^b^	9.58^c^	9.62^c^	11.32	
Methylamine (mM)									
12	0.33	0.30	0.32	0.33	0.30	0.29	0.31	0.31^y^	<0.001
24	0.33	0.34	0.31	0.33	0.34	0.32	0.30	0.33^y^	
48	0.33	0.34	0.34	0.32	0.34	0.35	0.49	0.36^x^	
Ethylamine (mM)									
12	0.39^ab^	0.33^b^	0.38^ab^	0.39^ab^	0.43^a^	0.30^b^	0.31^b^	0.36	<0.001
24	0.46	0.36	0.34	0.47	0.41	0.38	0.39	0.40	
48	0.35^c^	0.36^c^	0.35^c^	0.35^c^	0.50^a^	0.42^b^	0.46^ab^	0.40	
Tyramine (mM)									
12	0.37	0.33	0.34	0.37	0.38	0.38	0.42	0.37^y^	<0.001
24	0.34	0.35	0.34	0.44	0.39	0.45	0.35	0.38^y^	
48	0.56	0.57	0.56	0.65	0.60	0.67	0.59	0.60^x^	
Total biogenic amines (mM)									
12	15.25^a^	13.09^ab^	12.05^b^	12.51^b^	11.67^b^	11.06^b^	10.71^b^	12.34^xy^	<0.001
24	15.86^a^	10.47^bc^	11.41^bc^	12.44^b^	12.08^bc^	11.13^bc^	10.41^c^	11.97^y^	
48	17.22^a^	10.62^c^	11.21^c^	12.83^b^	14.67^b^	11.02^c^	11.17^c^	12.68^x^	

GABA, γ-aminobutyric acid; GPB, GABA-producing bacteria.

1) Con, 0 g/L GPB; T1, 2 g/L GPB; T2, 5 g/L GPB; T3, 2 g/L autoclaved GPB; T4, 5 g/L autoclaved GPB; T5, 2 g/L GABA; and T6, 5 g/L GABA.

Different superscript letters within rows (^a–c^) and columns (^w,y^) indicate significant differences at the 5% level, as indicated by Duncan’s multiple range test.

**Table 5:** Effect γ-aminobutyric acid on the performance of Hanwoo steers

Parameter	Treatment1)			Mean	SEM	p-value

	Control	T1	T2			
Weight gain (kg)	91.33	97.83	109.67	99.61	7.19	0.247
Average daily gain (kg)	0.71	0.76	0.85	0.77	0.05	0.238
Feed conversion ratio (kg feed/kg gain)	13.7	12.47	11.42	12.53	0.94	0.280
Marbling score	6.17	7.17	5.50	6.28	0.62	0.199
Blood endotoxins (U/mL)	29.23^a^	17.23^b^	16.42^b^	20.96	2.66	0.025

SEM, standard error of the mean; GABA, γ-aminobutyric acid; GPB, GABA-producing bacteria.

1) Control: 0 g/L GPB, T1: 2 g/L GPB, T2: 5 g/L GPB.

a,b Different superscript letters within rows indicate significant differences at the 5% level, as indicated by Duncan’s multiple range test.
